# Case Report: Case analysis of a juvenile type 1 diabetes mellitus patient with Mauriac syndrome

**DOI:** 10.3389/fendo.2026.1858339

**Published:** 2026-07-15

**Authors:** Rong Fu, Mengmeng Yang, Yanyan Guo, Yiyun Huang, Xiaoze Li, Mingying Zhang

**Affiliations:** 1 Children's Hospital, Tianjin University/Tianjin Children's Hospital, Tianjin Key Laboratory of Birth Defects for Prevention and Treatment, Tianjin, China; 2 School of Sports and Health, Tianjin Institute of Physical Education, Tianjin, China

**Keywords:** diabetic ketoacidosis type 1, glycogenic hepatopathy, hereditary hemochromatosis, lactic acidosis, Mauriac syndrome

## Abstract

**Medical history summary:**

A 14-year-old female with type 1 diabetes for over six years presented with abdominal pain for two days and vomiting for three hours. Her regimen includes NovoRapid before meals and glargine at bedtime, but she has irregular meal patterns, inconsistent blood glucose monitoring, and poor insulin adherence. She has a history of recurrent diabetic ketoacidosis (DKA) and related hospitalizations.

**Clinical presentation:**

The patient is alert but mildly weak, with adequate responsiveness. Breathing is slightly increased, deep, and regular. Extremities are cool. Radial pulses are strong, but dorsalis pedis pulses are diminished bilaterally. There is scattered right abdominal tenderness. The liver is palpable 5 cm below the costal margin, with medium texture and blunt borders, and percussion tenderness is positive.

**Diagnostic methods:**

Venous blood glucose was 21.86 mmol/L, and HbA1c was 10%. Arterial blood gas showed pH 6.933, pCO2 31.4 mmHg, and BEb -25.3 mmol/L. Insulin was below 1.39 pmol/L, and C-peptide below 0.003 pmol/L. Urinalysis revealed glucose 4+ and ketones 1+, leading to a diagnosis of type 1 DKA. Liver function was abnormal, with hepatomegaly and blood lactate at 9.85 mmol/L. Metabolic, infectious, and immune causes were excluded. Liver biopsy confirmed glycogenic hepatopathy, establishing a diagnosis of Mauriac syndrome with lactic acidosis. Serum uric acid ranged from 434 to 545 mmol/L, indicating hyperuricemia. Whole-exome sequencing identified a homozygous HFE mutation, suggesting hereditary hemochromatosis, though ferritin was normal and no iron deposition was seen on pathology.

**Treatment:**

Continuous intravenous insulin therapy was initiated, along with diabetes education, dietary guidance, exercise, glucose monitoring, and symptomatic care. An insulin pump was subsequently used for continuous subcutaneous insulin infusion (with Gansulin R before meals). Later, the pump was discontinued and switched to a four-injection subcutaneous regimen, consisting of NovoRapid before meals and insulin glargine at bedtime. Bicyclol Tablets was given for hepatoprotection.

**Clinical outcome:**

The patient was hospitalized for 13 days, remained afebrile, and reported no discomfort. Blood glucose ranged from 3.1 to 15.2 mmol/L. Liver size slightly decreased, but tenderness persisted. Liver function and lipids were mostly normal at discharge. Two months later, she was readmitted for DKA; liver function was normal, but hepatomegaly with tenderness persisted.

## 1 Introduction

Mauriac syndrome, also referred to as Glycogenic Hepatopathy (GH), represents a rare complication associated with inadequately managed type 1 diabetes mellitus (T1DM), and is sometimes termed diabetic pseudodwarfism. This syndrome primarily manifests in pediatric and adolescent populations with diabetes. Its characteristic clinical features (1) include elevated transaminase levels, hepatomegaly, short stature, delayed puberty, Cushingoid features, and hypercholesterolemia. Additional symptoms may encompass abdominal pain, vomiting, and increased lactate levels. The pathophysiological mechanisms underlying this syndrome are multifaceted, involving factors such as inappropriate insulin therapy, poor glycemic control, abnormal hepatic glycogen metabolism, suppressed growth hormone secretion, and lipid metabolism disorders. Despite its low incidence, Mauriac syndrome has a significant long-term impact on the health of affected children. Furthermore, its clinical presentation is heterogeneous and can be easily mistaken for other conditions. Consequently, accurate diagnosis necessitates a comprehensive approach, integrating medical history, physical examination, laboratory tests, and imaging studies.

This article delineates the diagnostic and therapeutic trajectory of a 14-year-old patient diagnosed with Mauriac syndrome, characterized by recurrent episodes of diabetic ketoacidosis and impaired liver function. A definitive diagnosis of glycogenic hepatopathy was achieved through histopathological evaluation of a liver biopsy, facilitating the timely identification of Mauriac syndrome and the initiation of an integrated treatment regimen. This case underscores the necessity for clinicians to meticulously monitor liver function, as well as growth and developmental parameters, in pediatric patients with a prolonged history of type 1 diabetes. In instances where children exhibit hepatomegaly, dyslipidemia, elevated lactate levels, and other related abnormalities, the potential for Mauriac syndrome should be considered, warranting prompt investigative measures to confirm the diagnosis. The objectives of this case report are to emphasize: (1) the complexities associated with the long-term management of adolescent patients with type 1 diabetes; (2) the identification and management strategies for Mauriac syndrome; (3) the significance of addressing multisystem involvement through comprehensive treatment approaches; (4)the clinical diagnosis and screening protocols for hereditary hemochromatosis.

## 2 Clinical data

### 2.1 General information

A 14-year-old female with a six-year history of type 1 diabetes mellitus (T1DM) presented with a two-day history of abdominal pain and three-hour history of vomiting. She had been prescribed insulin aspart (18–20 IU before meals) and insulin glargine (22 IU at bedtime), but demonstrated poor adherence to meal schedules, blood glucose monitoring, and insulin administration. Two days prior to admission, she experienced the onset of abdominal pain, followed by vomiting three hours prior to presentation. Upon arrival, her capillary blood glucose level was measured at 28.4 mmol/L, leading to her admission with diabetic ketoacidosis (DKA). Since her initial diagnosis in October 2018, she has experienced multiple hospitalizations due to DKA. During her 12th hospitalization in January 2025, hepatomegaly was observed, with the liver palpable 7 cm below the costal margin, alongside abnormal liver function tests, which normalized 3–5 days post-discharge. She has no history of infectious diseases, surgical interventions, trauma, or blood transfusions, and no known allergies. She was delivered full-term via cesarean section and achieved normal developmental milestones. Menarche occurred at age 12 with regular menstrual cycles, although she experienced amenorrhea from October 2024 to February 2025. Both parents are reported to be in good health.

### 2.2 Examination

Auxiliary Examinations: Auxiliary tests revealed multiple abnormalities. Complete blood count showed marked leukocytosis (WBC 22.51 × 10^9^/L; normal 3.5–9.5 × 10^9^/L) with elevated hemoglobin and hematocrit. Arterial blood gas analysis indicated severe metabolic acidosis: pH 6.933, pCO_2_ 31.4 mmHg, BE -25.3 mmol/L, and HCO_3_^-^ 6.6 mmol/L. Urinalysis detected glucose (4+) and ketones (1+). Biochemistry revealed hyperglycemia (venous glucose 21.86 mmol/L; normal 3.9–6.1 mmol/L), while electrolytes and amylase were normal.Endocrine assessment showed insulin levels below 1.39 pmol/L (normal range: 5–25 pmol/L) and C-peptide levels below 0.003 pmol/L (normal range: 0.3-1.3 nmol/L), with an HbA1c of 10% (normal range: 4%-6%). Liver function tests indicated elevated levels of alanine aminotransferase (ALT) at 124 U/L (normal range: 7–40 U/L), aspartate aminotransferase (AST) at 41 U/L (normal range: 13–35 U/L), and gamma-glutamyl transferase (GGT) at 53 U/L (normal range: 7–32 U/L). The lipid profile revealed elevated triglycerides (TG) at 6.29 mmol/L (normal range: 0.56-1.7 mmol/L) and total cholesterol (TC) at 6.78 mmol/L (normal range: 3.1-5.7 mmol/L). Finally, lactate levels were markedly elevated at 9.85 mmol/L, compared to the normal range of 0.5-1.6 mmol/L, suggesting lactic acidosis.

**Figure 1:**
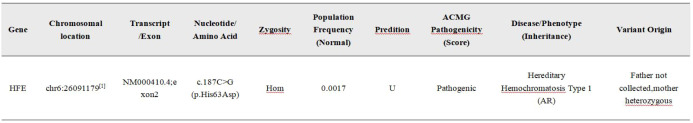
HFE gene analysis report. The analysis of this sample identifies a homozygous mutation in the HFE gene, characterized by a cytosine-to-guanine substitution at nucleotide position 187 (c.187C>G). This genetic alteration leads to the replacement of histidine with aspartic acid at amino acid position 63 (p.His63Asp). Based on the ACMG guidelines, this variant is provisionally classified as Pathogenic, supported by criteria PS3 and PS4. Criterion PS3 is met as functional studies indicate that this variant impacts gene or protein function, while criterion PS4 is satisfied due to the significantly higher prevalence of this variant in affected individuals compared to the general population.

Additional Diagnostic Assessments: Cortisol, ACTH, and sex hormone levels were within normal ranges. Thyroid Function Tests: Triiodothyronine (T3) measured at 1.19 nmol/L, which is below the normal reference range of 1.3-3.1 nmol/L, while other thyroid parameters were normal. Hemolysis evaluation returned negative. Screening for viral hepatitis, autoimmune liver disease antibodies, alpha-fetoprotein, and ceruloplasmin revealed no abnormalities. Tests for inherited metabolic disorders and urine organic acids were unremarkable. Genetic Analysis: Whole Exome Sequencing identified a homozygous mutation in the HFE gene (refer to **Figure 1**). Functional assays demonstrate that this variant impairs gene/protein function and is strongly correlated with Hereditary Hemochromatosis Type 1.

**Figure 2:**
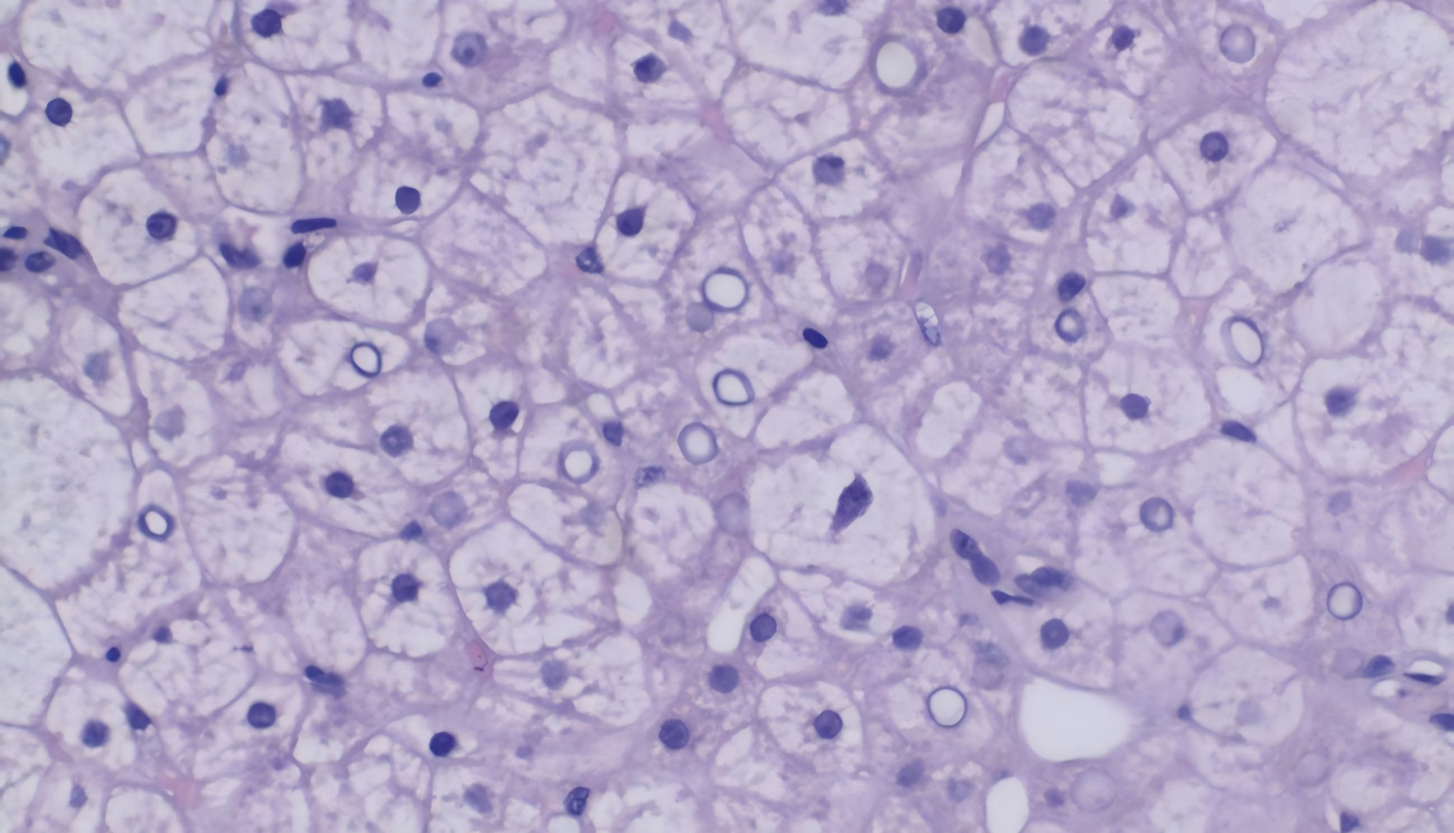
Liver biopsy pathology results. Liver Biopsy Report: A single core of liver biopsy tissue, measuring 1.8 cm in length and 0.1 cm in diameter, was submitted for histological examination. Microscopic analysis reveals pronounced abnormalities within the liver tissue. There is a diffuse and marked swelling of hepatocytes, resulting in the compression of hepatic sinusoids. The cytoplasm of the hepatocytes appears pale and clear, with small, centrally located nuclei. A minor degree of nuclear vacuolar degeneration is present. Due to prior processing effects, cytoplasmic PAS staining shows partial positivity, which is eliminated following diastase digestion. There is no significant inflammatory infiltration within the lobules or portal areas. The interlobular bile ducts are intact, and the limiting plate remains unaffected. Mild proliferation of collagen fibers is observed, accompanied by localized mild perisinusoidal fibrosis, although no fibrous septa are detected. Preliminary Diagnosis: Type 1 Diabetes Mellitus with Ketoacidosis; Acute Gastroenteritis; Pubertal Development (Tanner Stage B4P3); Lactic Acidosis; Hyperuricemia; Hepatomegaly of Undetermined Etiology (Pending Further Investigation).

Additional Examinations: Ultrasound imaging indicated hepatomegaly, with the upper border reaching the 5th intercostal space, subxiphoid measurement at 69 mm, and subcostal measurement at 60 mm. The liver capsule appeared smooth with a blunt edge, and the parenchymal echotexture was homogeneous, slightly enhanced, and coarse. The left lobe extended into the left hypochondriac region, and the common bile duct measured approximately 5 mm in width. Computed Tomography (CT) Scan: Demonstrated an enlarged liver morphology without any abnormal density lesions within the liver parenchyma. Liver Biopsy Pathology (refer to **Figure 2**): Findings were consistent with Glycogenic Hepatopathy, indicative of Mauriac Syndrome.

### 2.3 Progress notes

#### 2.3.1 Initial progress note

**Figure 3:**
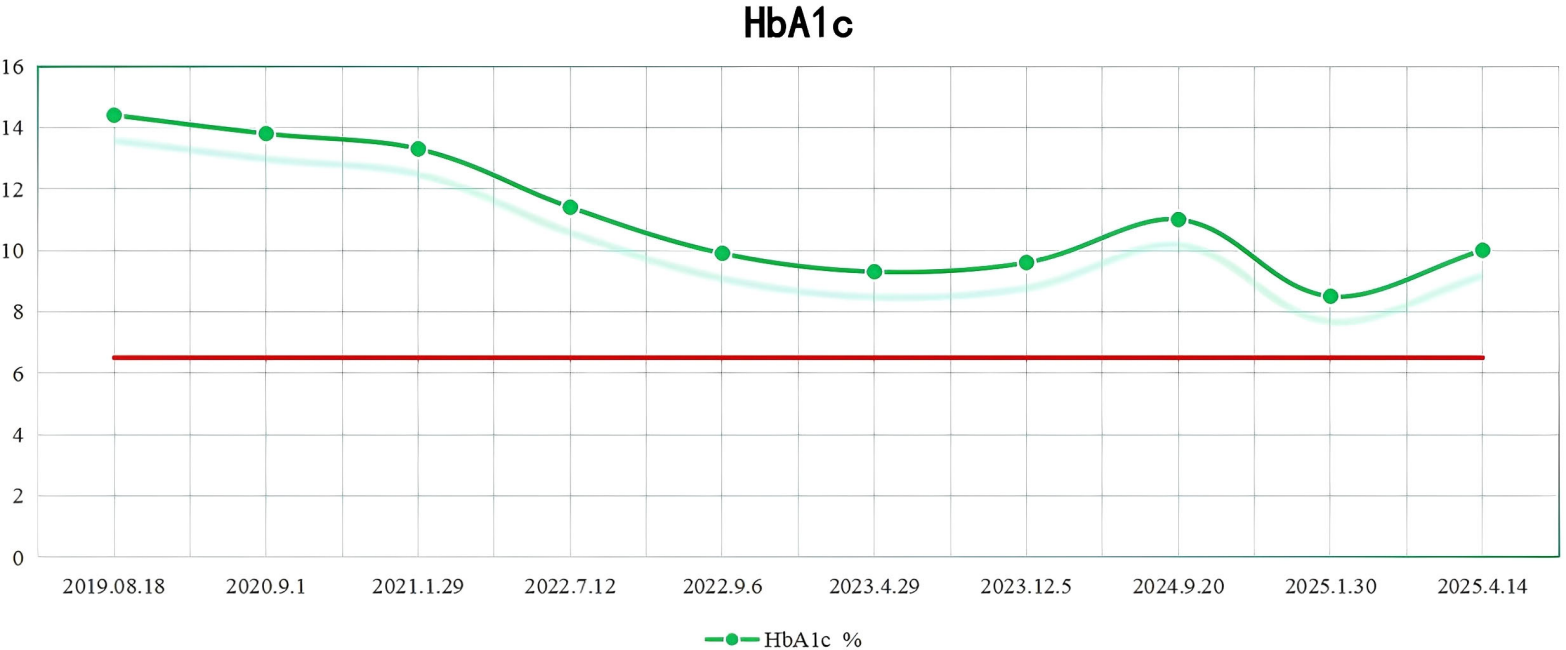
Patient’s usual HbA1c trend.

**Figure 4:**
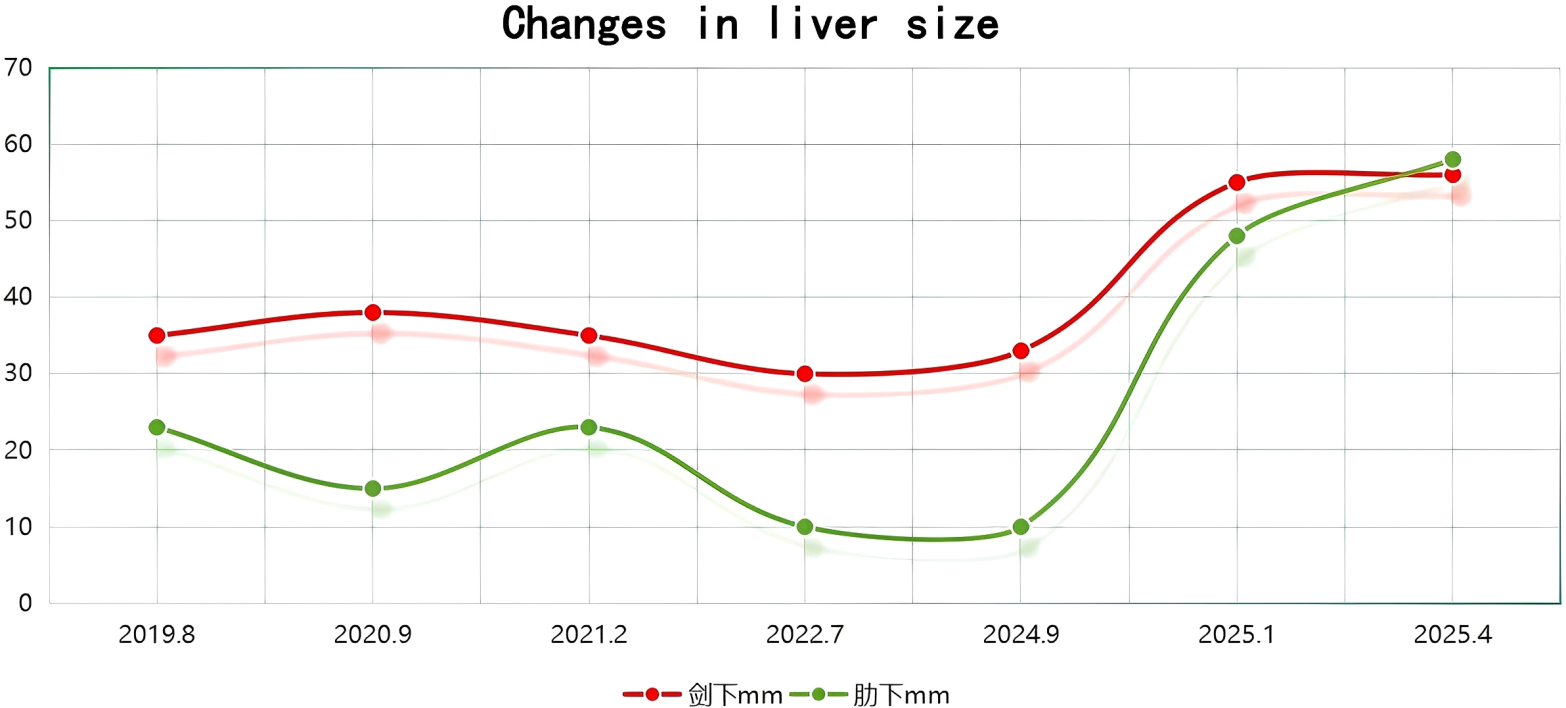
Changes in patient’s liver size over time.

**Figure 5:**
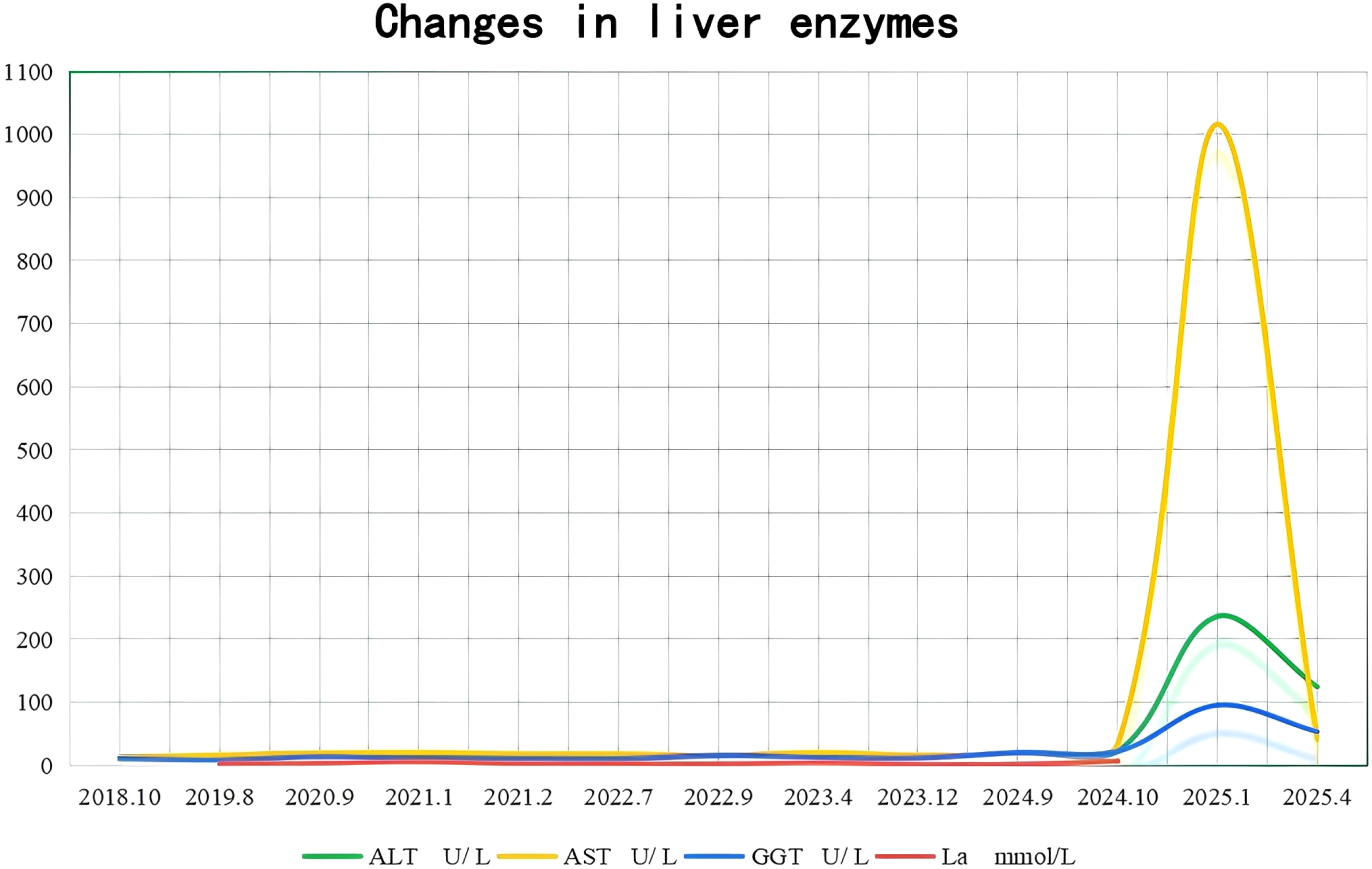
Changes in patient’s liver enzymes over time.

Diagnosis and Diagnostic Basis:

1. Type 1 Diabetes Mellitus with Ketoacidosis: The patient, with a documented history of Type 1 Diabetes Mellitus, was admitted due to symptoms of abdominal pain and vomiting. Laboratory investigations revealed a blood glucose concentration of 21.86 mmol/L, acidosis as indicated by blood gas analysis, and the presence of glucose and ketones in the urine. Additionally, insulin and C-peptide levels were found to be markedly low. Consequently, a diagnosis of Type 1 Diabetes Mellitus with Ketoacidosis was confirmed.
2. Mauriac Syndrome: The patient presents with a documented history of inadequate long-term glycemic control and numerous prior hospitalizations due to diabetic ketoacidosis. Since the initial diagnosis, the patient’s HbA1c levels have persistently exceeded therapeutic targets (refer to **Figure 3**). During the patient’s 12th hospitalization for abdominal pain, hepatomegaly was first observed, characterized by an increased and coarse echotexture of the liver parenchyma. Liver dimensions were measured at 67 mm below the xiphoid process and 66 mm below the costal margin. Liver enzyme levels were markedly elevated: ALT at 236 U/L, AST at 1016 U/L, and GGT at 95 U/L, while ferritin levels remained within normal range. Post-treatment, there was an improvement in hepatomegaly, with measurements reducing to 55 mm subxiphoid and 48 mm subcostal. Upon the current admission, physical examination continues to reveal hepatomegaly with associated hepatic tenderness and percussion tenderness. Abnormal liver function persists (refer to **Figures 4**, **5**). A liver biopsy pathology consultation confirmed findings consistent with Glycogenic Hepatopathy, leading to a diagnosis of Mauriac Syndrome (1).
3. Pubertal Development: The patient, a 14-year-old female, has been evaluated and classified as Tanner Stage B4P3, leading to a diagnosis of advanced pubertal development.
4. Lactic Acidosis: The patient’s blood lactate concentration is markedly elevated, and blood gas analysis reveals acidosis, resulting in a diagnosis of lactic acidosis.
5. Hyperuricemia: The patient’s serum uric acid concentration is significantly elevated, warranting a diagnosis of hyperuricemia.

Differential Diagnosis:

1. Glycogen Storage Disease (GSD): Glycogen Storage Disease comprises a group of inherited disorders affecting glycogen metabolism. The clinical manifestations typically include fasting hypoglycemia, lactic acidosis, hepatomegaly and/or nephromegaly, hyperlipidemia, hypercholesterolemia, hyperuricemia, and growth retardation. Notably, individuals with GSD generally do not present with a history of diabetes mellitus. In this case, the patient’s clinical presentation does not correspond with the hallmark symptoms of Glycogen Storage Disease, thereby rendering a diagnosis of GSD unlikely (2).
2. Hereditary Hemochromatosis (HH): The diagnostic criteria for Hereditary Hemochromatosis are as follows:① Elevated serum iron indices; ② Evidence of iron overload in the liver and/or spleen as observed on MRI, or the presence of iron deposition confirmed by liver histopathology; ③ Exclusion of secondary causes of iron overload; ④ Identification of a pathogenic variant in a gene associated with iron metabolism.The diagnostic process is multifaceted and cannot be based on a single criterion. In this case, although genetic analysis identified a homozygous mutation in the HFE gene, this finding only fulfills the fourth diagnostic criterion. Therefore, the hepatomegaly and hepatic dysfunction attributable to HH are not substantiated, and the child is currently considered to be in a phase of genetic susceptibility (3).
3. Viral Hepatitis: Extensive hepatitis panel testing for this patient yielded negative results. Additionally, pathogen assessments for TORCH viruses, Epstein-Barr virus (EBV), Mycoplasma pneumoniae (MP), and tuberculosis (TB) demonstrated no abnormalities. Consequently, the diagnosis of Viral Hepatitis is not substantiated.
4. Autoimmune Hepatitis (AIH):The patient tested positive for Antinuclear Antibodies (ANA); however, within the Autoimmune Liver Disease Panel 1, only ANA with a nuclear granular pattern was positive, while all other markers were negative. Moreover, a 15-item ANA profile was entirely negative. In the absence of additional typical clinical manifestations of Autoimmune Hepatitis, this diagnosis is not supported.
5. Non-Alcoholic Fatty Liver Disease (NAFLD):Non-Alcoholic Fatty Liver Disease (NAFLD) exhibits clinical manifestations that are comparable to those of Glycogenic Hepatopathy (GH) and represents the most prevalent liver disease among individuals with diabetes mellitus. Nevertheless, the pathogenesis and progression of NAFLD are closely linked to obesity and insulin resistance, primarily manifesting in the context of Type 2 Diabetes Mellitus, with a potential progression to liver failure and cirrhosis (4). This clinical profile does not align with the patient’s medical history, thereby rendering a diagnosis of NAFLD unsupported.

Treatment Plan:

1. Treatment Principles and Rationale: The fundamental principles of management focus on achieving glycemic control, correcting acidosis, implementing hepatoprotective therapy, and addressing complications, while simultaneously enhancing patient and family education to improve adherence to treatment. In light of the patient’s current condition of Diabetic Ketoacidosis and abnormal liver function, immediate priority is given to managing life-threatening complications, followed by a gradual optimization of the long-term treatment strategy.
2. Treatment Regimen and Specific Measures: Upon admission, the patient received continuous intravenous insulin infusion with close monitoring of blood and urine glucose levels. After stabilization, therapy was transitioned to continuous subcutaneous insulin infusion via an insulin pump using Gansulin R, with dose adjustments based on glycemic profiles. Prior to discharge, the regimen was converted to subcutaneous injections of insulin aspart before meals and insulin glargine at bedtime.

Supportive management included hepatoprotective therapy with bicyclol, correction of fluid, electrolyte, and acid–base imbalances, and monitoring and management of lactate levels. During hospitalization, a liver biopsy was performed to clarify hepatic pathology. Serial reassessment of complete blood count, liver function, lipid profile, lactate, blood glucose, and urine ketones was conducted. Follow-up abdominal ultrasound was used to evaluate liver status, and genetic testing for inherited metabolic disorders was completed. Patient education, including diabetes management, dietary guidance, and adherence to medication and glucose monitoring, was also provided.

#### 2.3.2 Additional progress notes

(1) April 14, 2025 (Hospital Day 1):

Upon admission, continuous intravenous insulin infusion was initiated with close monitoring of blood and urine glucose. By evening, mental status improved, and oral intake resumed. Therapy was transitioned to continuous subcutaneous insulin infusion via an insulin pump using Gansulin R. Hepatoprotective therapy with bicyclol was started.

(2) April 17, 2025 (Hospital Day 4):

Ultrasound-guided percutaneous liver biopsy was performed. Liver function tests improved (ALT 37 U/L, AST 27 U/L, GGT 33 U/L), and lactate decreased to 3.48 mmol/L. Current management was continued.

(3) April 19, 2025 (Hospital Day 6):

Urinary ketones resolved. The insulin pump was discontinued, and therapy was switched to subcutaneous NovoRapid (12/10/14 IU before meals) plus insulin glargine (24 IU at bedtime). Supportive treatment was continued.

(4) April 20–22, 2025 (Hospital Days 7–9):

Blood glucose was continuously monitored, and insulin doses were adjusted accordingly. Liver function and lipid profiles showed marked improvement. Dietary and exercise guidance was reinforced.

(5) April 23, 2025 (Hospital Day 10):

Follow-up ultrasound showed persistent hepatomegaly with heterogeneous, coarse echotexture (56 mm subxiphoid, 58 mm subcostal). Glycemic monitoring and insulin adjustments were continued alongside dietary management. Complete blood count and venous blood gas results are presented in **Tables 1** and **2**.

**Table 1:** Complete blood count results during hospitalization.

Complete blood count	4.14 (6:21)	4.14 (16:31)	4.17	4.18	4.20
Hb (114-154)g/L	168	144	124	117	117
HCT (36-47)%	54.8	43.9	38.6	36.3	37.6
WBC (4-11)10*9/L	22.51	13.84	5.04	4.36	4.47
N%	76	72	28	53	51
L%	20	23	66	41	42
M%	4	5	4	4	5
E%			2	2	2
RET %(0.5-1.5)		3.13	2.03		
PLT (150-407)10*9/L	554	420	250	259	298
CRP (0-8)mg/L	5.27	4.52	<2.5	<2.5	<2.5

**Table 2:** Venous blood gas results during hospitalization.

Venous blood gas	4.14 (6:21)	4.14 (16:31)	4.15	4.17
PH (7.32-7.42)	6.933	7.285	7.3	7.393
pC02 (41-45)mmHg	31.4	23.9	36.7	46.7
Beb mmol/L	-25.3	-13.3	-7.7	2.6
HCO3^-^ (21-28)mmol/L	6.6	11.4	18	28.4

#### 2.3.3 Discharge summary

A 14-year-old female with a history of Type 1 Diabetes Mellitus exceeding six years was admitted due to diabetic ketoacidosis, hyperglycemia (21.86 mmol/L), abnormal liver function, and hepatomegaly. During her hospitalization, glycemic control was achieved initially through intravenous insulin administration, followed by subcutaneous insulin therapy, which included NovoRapid and insulin glargine. Hepatoprotective and supportive treatments were also administered, resulting in clinical improvement. Upon discharge, the patient was in stable condition, exhibiting improved liver function, negative urine ketones, and fluctuating but generally controlled blood glucose levels. The final diagnoses included Type 1 Diabetes Mellitus with ketoacidosis, Mauriac syndrome, lactic acidosis, and hyperuricemia. The patient was advised to continue the insulin regimen, consisting of preprandial NovoRapid and bedtime insulin glargine, perform regular blood glucose monitoring, adhere to a diabetic diet and engage in appropriate physical activity, and maintain hepatoprotective therapy. Regular follow-up for monitoring metabolic and hepatic parameters was recommended.

#### 2.3.4 Additional case information

There were no occurrences of inter-hospital transfers, intra-departmental transfers, or mortality-related events. Throughout the hospitalization period, the complexity of the diagnosis, which involved multidisciplinary considerations in the fields of Endocrinology and Gastroenterology, necessitated a thorough evaluation and investigation. Consequently, the diagnosis was refined, and a targeted treatment plan was subsequently implemented.

### 2.4 Follow-up and outcome

The management of pediatric diabetes necessitates continuous dedication, financial resources, and educational support; deficiencies in any of these areas can result in suboptimal patient adherence and the onset of complications (5). Post-discharge monitoring of the patient’s treatment regimen indicated that insulin administration was inconsistent, and the patient did not comply with prescribed meal schedules and blood glucose monitoring. Two months subsequent to discharge, the patient was readmitted due to another episode of Diabetic Ketoacidosis. At that time, liver function tests were within normal limits; however, hepatomegaly accompanied by tenderness persisted. A liver ultrasound revealed dimensions of 58 mm below the xiphoid process and 58 mm below the costal margin, with a heterogeneous, coarse parenchymal echotexture and patchy areas of increased echogenicity—findings that remained largely unchanged from the previous hospitalization.

## 3 Discussion

This case report delineates the diagnostic and therapeutic journey of a 14-year-old patient diagnosed with Type 1 Diabetes Mellitus, further complicated by Mauriac Syndrome and multiple systemic abnormalities. The clinical intricacy is chiefly reflected in the challenges associated with etiological diagnosis and adherence to treatment protocols. The ensuing discussion addresses these critical issues in conjunction with pertinent literature.

### 3.1 Recurrent nature of diabetic complications in adolescents

Mauriac Syndrome, also referred to as Glycogenic Hepatopathy (GH), represents a rare complication linked to poorly managed Type 1 Diabetes Mellitus (T1DM). It is alternatively known as diabetic pseudodwarfism and predominantly affects children and adolescents with diabetes. The syndrome is characterized by clinical features such as elevated transaminases, hepatomegaly, short stature, delayed puberty, Cushingoid features, and hypercholesterolemia. Additional manifestations may include abdominal pain, vomiting, and elevated lactate levels (6).Mauriac Syndrome is recognized as a potentially reversible condition. In this particular case, the patient exhibited recurrent episodes of Diabetic Ketoacidosis, attributable to chronically suboptimal glycemic control. Since her initial diagnosis, the patient has not consistently adhered to structured meal schedules, routine blood glucose monitoring, or regular insulin administration. This non-adherence has resulted in significant glycemic variability, thereby increasing her susceptibility to ketoacidosis. Additionally, the patient is undergoing puberty, a developmental stage characterized by hormonal changes that can negatively impact glycemic control and elevate the risk of Diabetic Ketoacidosis. Numerous studies have demonstrated that managing glycemic levels in adolescents with diabetes presents unique challenges due to hormonal fluctuations and suboptimal treatment adherence, leading to a heightened incidence of ketoacidosis (7). Effective long-term disease management necessitates a high degree of compliance; in this patient’s situation, psychological or familial factors may have contributed to the difficulty in maintaining strict adherence to the treatment regimen. Consequently, early screening, diagnosis, and intervention are essential to prevent the onset and progression of severe complications.

### 3.2 Diagnostic algorithm for mauriac syndrome and its impact on patients

In individuals with Type 1 Diabetes Mellitus exhibiting poor glycemic control and presenting with moderate to severe elevations in transaminases—particularly with an aspartate aminotransferase (AST)-predominant pattern—alongside a hepatocellular or mixed pattern of liver injury, the potential diagnosis of Glycogenic Hepatopathy (GH) should be contemplated. Abdominal ultrasound and blood tests are instrumental in excluding alternative etiologies of liver disease. Non-contrast computed tomography (CT) or dual-echo magnetic resonance imaging (MRI) may yield indicative evidence of GH. Upon suspicion of GH, the implementation of stringent glycemic control is advised. Should weekly liver function tests reveal substantial improvement or normalization, such findings corroborate the diagnosis of GH. Conversely, if abnormalities in liver function tests persist despite enhanced glycemic management, consideration should be given to performing a liver biopsy. This diagnostic strategy may facilitate the avoidance of unnecessary liver biopsies in certain patients with Type 1 Diabetes Mellitus who are suspected of having GH (8).

The extant literature suggests a strong correlation between Mauriac Syndrome and prolonged inadequate glycemic control. Recurrent episodes of Diabetic Ketoacidosis constitute a significant risk factor for the onset of growth hormone (GH) abnormalities. The pathophysiological mechanism underlying this condition involves the excessive accumulation of glycogen within hepatocytes, which subsequently disrupts hepatic function and lipid metabolism. As an infrequent complication of Type 1 Diabetes Mellitus, Mauriac Syndrome can result in growth retardation, hepatic dysfunction, and dyslipidemia. Therefore, vigilant monitoring for potential complications is crucial in patients with Type 1 Diabetes Mellitus, and prompt diagnosis and intervention are critical (9).

### 3.3 Homozygous HFE gene mutation

The patient with a homozygous HFE gene mutation has not exhibited any clinical manifestations of disease. Current literature suggests that a homozygous HFE gene mutation does not necessarily result in the development of Hereditary Hemochromatosis (HH^)^ (10). While homozygous HFE mutations are the primary genetic determinant of HH, the manifestation of the disease is also modulated by additional genetic and environmental factors. For example, an individual’s broader genetic makeup, lifestyle choices, and dietary practices can influence the expression of the disease. Moreover, even in cases of homozygous mutation, there may be instances of incomplete penetrance or a delayed onset of the condition.

Moreover, mutations in the HFE gene can contribute to the development of secondary diabetes mellitus and liver damage. Homozygous mutations in HFE result in enhanced intestinal iron absorption and a reduced capacity for hepatic iron storage, culminating in excessive iron accumulation within the body. This iron overload induces oxidative stress and subsequent tissue injury. Simultaneously, excessive iron levels can disrupt insulin signaling pathways, leading to insulin resistance and impaired glycemic regulation. Chronic elevation of iron levels may also inflict damage on pancreatic β-cells, thereby compromising their insulin secretory capacity and further exacerbating the progression of diabetes mellitus. The liver, as the primary organ for iron storage, experiences increased iron deposition due to overload, leading to hepatocyte injury and inflammatory responses. Persistent hepatocyte damage and inflammation can activate hepatic stellate cells, promoting the synthesis and deposition of collagen fibers, which ultimately results in hepatic fibrosis and potentially progresses to cirrhosis.

It is crucial to acknowledge that although the clinical manifestations potentially arising from a homozygous HFE gene mutation may mimic those observed in Mauriac Syndrome, the underlying mechanisms are fundamentally different. Mauriac Syndrome is a disorder marked by growth retardation and hepatosplenomegaly linked to insulin deficiency, predominantly seen in children with Type 1 Diabetes Mellitus. Its pathogenesis is markedly distinct from the secondary diabetes and hepatic damage caused by homozygous HFE gene mutations.

### 3.4 Pubertal development and diabetes management

Variations in sex hormone levels during puberty can significantly impact insulin sensitivity and glycemic regulation mechanisms (11). Adolescent patients often demonstrate decreased adherence to treatment regimens due to factors such as increased self-consciousness and psychological reactance or rebelliousness. Simultaneously, the nutritional demands escalate during this phase of rapid growth and development, necessitating modifications to dietary plans to satisfy growth requirements while ensuring effective glycemic control. Current research underscores that managing diabetes during adolescence necessitates a holistic approach that integrates physiological, psychological, and social dimensions, advocating for individualized treatment strategies and enhancing patient education and psychological support (12).

### 3.5 Type 1 diabetes mellitus in other rare syndromes such as Moyamoya disease

Beyond Mauriac syndrome, the interaction between poorly controlled type 1 diabetes mellitus (T1DM) and other rare systemic disorders warrants clinical attention. Moyamoya disease, a progressive cerebrovascular disorder characterized by stenosis of the internal carotid arteries and the development of fragile collateral vessels (exhibiting a ‘cloud of smoke’ appearance), has increasingly been observed to co-occur with autoimmune conditions, including T1DM (13). Although Moyamoya disease primarily manifests as ischemic or hemorrhagic strokes in children and young adults, its association with T1DM introduces significant diagnostic considerations. Patients with long-standing T1DM who exhibit unexplained neurological symptoms—such as transient ischemic attacks, persistent headaches, seizures, or acute focal deficits—should be assessed for potential Moyamoya disease, especially when suboptimal glycemic control does not fully explain the neurological manifestations. Conversely, in patients diagnosed with Moyamoya disease, it may be prudent to screen for autoimmune endocrinopathies, including T1DM, as both conditions may share underlying genetic or immune-mediated mechanisms. From a clinical perspective, this dual consideration is essential for comprehensive patient management.

### 3.6 Limitations

The limitations of this case report are as follows: ① The genetic testing conducted was restricted to genes already associated with known diseases, thereby excluding genes that have not yet been fully characterized. ② While it is acknowledged that the patient did not maintain regular meal schedules, consistent blood glucose monitoring, or regular insulin administration, the report does not offer an in-depth analysis of strategies to enhance patient adherence. ③Relevant parental information was not documented in the original case records, which limits the assessment of genetic and growth-related contributions to the patient’s clinical presentation.④The report lacks a comprehensive account of the patient’s long-term follow-up status post-discharge. Long-term follow-up is essential for assessing treatment efficacy, monitoring disease progression, and adjusting therapeutic regimens.

## 4 Conclusion

This case pertains to a 14-year-old female patient with a history of multiple hospitalizations for ketosis or diabetic ketoacidosis following her diagnosis with type 1 diabetes mellitus in 2018. In April 2025, she was admitted with symptoms of abdominal pain and vomiting, leading to a diagnosis of type 1 diabetic ketoacidosis, acute gastroenteritis, Mauriac syndrome, among other conditions. Her condition improved following the administration of insulin therapy, hepatoprotective treatment, and symptomatic management, allowing for her subsequent discharge. This case underscores several critical considerations: (1) Adolescent patients with diabetes mellitus necessitate enhanced glycemic management and vigilant monitoring for complications arising from prolonged hyperglycemia and immune dysregulation; (2) The importance of recognizing and differentiating Mauriac syndrome; (3) The clinical diagnosis and screening for hereditary hemochromatosis; (4) The necessity of multidisciplinary collaboration and comprehensive treatment to enhance prognosis. Future endeavors should prioritize the holistic assessment of the patient during treatment and the collection of long-term follow-up data to refine diagnostic and therapeutic approaches.

## Data Availability

The original contributios presented in the study are included in the article/ supplementary material. Further inquiries can be directed to the corresponding author.
